# Assessment of Gastroenteric Viruses in Marketed Bivalve Mollusks in the Tourist Cities of Rio de Janeiro, Brazil, 2022

**DOI:** 10.3390/v16030317

**Published:** 2024-02-20

**Authors:** Carina Pacheco Cantelli, Guilherme Caetano Lanzieri Tavares, Sylvia Kahwage Sarmento, Fernanda Marcicano Burlandy, Tulio Machado Fumian, Adriana Gonçalves Maranhão, Emanuelle de Souza Ramalho Ferreira da Silva, Marco Aurélio Pereira Horta, Marize Pereira Miagostovich, Zhihui Yang, José Paulo Gagliardi Leite

**Affiliations:** 1 Laboratory of Comparative and Environmental Virology, Oswaldo Cruz Institute, Fiocruz 21040-360, RJ, Brazil; 2 Multi-User Platform, Oswaldo Cruz Institute, Fiocruz 21040-360, RJ, Brazil; 3 Division of Molecular Biology, Office of Applied Research and Safety Assessment, Center for Food Safety and Applied Nutrition, U.S. Food and Drug Administration, Laurel, MD 20723, USA

**Keywords:** gastroenteric viruses, bivalve mollusk, genotyping, monitoring, ISO 15216, food safety

## Abstract

This study investigated the prevalence and genetic diversity of gastroenteric viruses in mussels and oysters in Rio de Janeiro, Brazil. One hundred and thirty-four marketed bivalve samples were obtained between January and December 2022. The viral analysis was performed according to ISO/TS 15216, and the screening revealed the detection of norovirus GII/GI (40.3%), sapovirus (SaV; 12.7%), human mastadenovirus (7.5%), and rotavirus A (RVA; 5.9%). In total, 44.8% (60) of shellfish samples tested positive for one or more viruses, 46.7% (28/60) of the positive samples tested positive for a single viral agent, 26.7% (16) tested positive for two viral agents, 8.3% (5) for three viral agents, and 13.3% (8) for four viral agents. Additionally, three mussel samples were contaminated with the five investigated viruses (5%, 3/60). Norovirus GII showed the highest mean viral load (3.4 × 10^5^ GC/g), followed by SaV (1.4 × 10^4^ GC/g), RVA (1.1 × 10^4^ GC/g), human mastadenovirus (3.9 × 10^3^ GC/g), and norovirus GI (6.7 × 10^2^ GC/g). Molecular characterization revealed that the recovered norovirus strains belonged to genotypes GII.2, GII.6, GII.9, GII.17, and GII.27; SaV belonged to genotypes GI.1 and GIV.1; RVA to genotypes G6, G8, P[8]-III, and human mastadenovirus to types F40 and F41. The GII.27 norovirus characterized in this study is the only strain of this genotype reported in Brazil. This study highlights the dissemination and diversity of gastroenteric viruses present in commercialized bivalves in a touristic area, indicating the potential risk to human health and the contribution of bivalves in the propagation of emerging pathogens.

## 1. Introduction

Food and water quality are considered essential drivers that may favor the emergence of pathogens and transmission of infectious agents through anthropic activities [1,2]. The shedding of foodborne viruses originates from the human gastrointestinal tract; their presence occurs through consumption or contact with sewage-contaminated water arising from the discharge of untreated or inadequately treated sewage into the aquatic environment, poor hygiene, or contamination by food handlers [3,4]. Studies have demonstrated that noroviruses, rotavirus A (RVA), and human mastadenoviruses are considered the most important aetiological agents of non-bacterial acute gastroenteritis (AGE) outbreaks transmitted by contaminated food and water [5,6]. Besides this, emerging viruses related to diarrheal diseases have been discovered recently, including sapoviruses (SaV) [7].

The presence of these viruses in wastewater is due significant to their high excretion from infected individuals, low removal in treatment processes, environmental persistence, and low infectious dose [8].

Bivalve mollusks can bioaccumulate and concentrate viruses from water in their digestive glands through their filter-feeding process [9,10]. The different frequencies of human viruses in bivalves may reflect their respective levels of circulation in the human population, concentration in stool and sewage, environmental persistence, or affinity for bivalve tissues [11]. AGE outbreaks caused by eating raw or insufficiently cooked norovirus-infected bivalves (oysters, mussels, cockles, and clams) are frequently reported worldwide [12,13,14].

Noroviruses and SaV belong to two separate genera of the family *Caliciviridae*, characterized to have a single-stranded, positive-sense genomic RNA of 6.4–8.5 kb organized into either two or three significant ORFs [15]. These nonenveloped viruses are highly infectious agents transmitted through various routes, including person-to-person contact with fecally contaminated food, water, or environmental surfaces. Noroviruses consist of a genetically diverse group that infects a wide range of mammalian host species, and, in humans, the genogroups GI and GII are the most reported non-bacterial agents related to foodborne outbreaks globally [16,17]. Based on the phylogenetic analysis of the complete VP1 gene, noroviruses can be further classified into genotypes, with at least nine genotypes belonging to GI and 26 to GII [18]. GII.4 variants predominate across all outbreak settings, though the role of GI and non-GII.4 genotypes appear to be greater in settings involving foodborne or waterborne transmission than GII.4 viruses [19,20]. In the context of norovirus molecular epidemiology, recombination has long been postulated to be a driving force of the emergence and re-emergence of different intragenotype recombinants [21]. SaV has been recognized as having at least 19 genogroups, among which GI, GII, GIV, and GV are classified as human strains and have very high genetic diversity; however, GI and GIV are the most common genogroups observed in AGE outbreaks [22,23,24]. According to the Global Pediatric Diarrhea Surveillance Network Study, SaV had the fourth-highest incident rate of viral AGE after the human mastadenovirus, noroviruses, and RVA [25].

RVA is a nonenveloped virus of the family *Reoviridae* belonging to the genus *Rotavirus*. The RVA genome is composed of 11 segments of double-stranded RNA, which encode six structural proteins (VP1-4, VP6, and VP7) and six non-structural proteins (NSP1-NSP6) [26]. The VP7 and VP4 genes of RVA are employed in the binary classification systems to delineate RVA into G (glycoprotein) and P (protease-sensitive) genotypes, respectively. Globally, to date, 42 G-genotypes and 58 P-genotypes have been described in different surveillance studies in both humans and animals (https://rega.kuleuven.be/cev/viralmetagenomics/virus-classification/rcwg, accessed on 12 January 2024). Clinically, the most common G/P genotype combinations causing 90% of infections in humans are G1P[8], G2P[4], G3P[8], G4P[8], G9P[8] and G12P[8] [17]. Moreover, due to the segmented genome, unusual genotypes have been detected and are usually related to interspecies transmission events that increase RVA genetic diversity [27,28].

Human mastadenovirus are nonenveloped viruses with a double-stranded DNA genome belonging to the *Mastadenovirus* genus in the *Adenoviridae* family. To date, 111 types of human mastadenoviruses have been characterized and further divided into seven species (A–G) (http://hadvwg.gmu.edu/, accessed on 10 November 2023). The usefulness of the human mastadenovirus as a virological water quality indicator is attributed to its environmental stability, wide distribution, and higher abundance relative to other enteric viruses, besides their easier detection than RNA viruses [29]. The clinical manifestations of human mastadenovirus infections are pervasive, such as respiratory infections, conjunctivitis, gastroenteritis, cystitis, myocarditis, cardiomyopathy, and meningoencephalitis [30,31]. Most AGE outbreaks of adenoviral enteric infections in humans are related to the human mastadenovirus species F40 and F41, with most cases registered in childhood [29]. During the first semester of 2022, the hypothesis that human mastadenovirus F41 may cause acute childhood hepatitis was investigated in several cases recorded in the United Kingdom, Europe, the United States, Argentina, and other countries [32]. Studies have demonstrated that human mastadenovirus F41 may play a role in these infections, but further investigation is necessary for a definitive explanation [33].

Data on the viral contamination of bivalves throughout the food chain, including points of sale, are scarce in Brazil. To contribute and best understand the epidemiological importance of those gastroenteric viruses contaminating seafoods, this study aimed to evaluate the occurrence and genetic diversity of gastroenteric viruses, including noroviruses, SaV, RVA, and human mastadenovirus, in commercialized bivalves (oysters and mussels) acquired over 12 months of 2022 in three crucial touristic coastal cities in the state of Rio de Janeiro, Brazil. The objective was to investigate the prevalence and diversity of these viruses to provide viral contamination data for further epidemiological studies to assess the health risks associated with bivalve mollusk consumption.

## 2. Material and Methods

### 2.1. Sample Collection and Viruses’ Recovery

**Figure 1:**
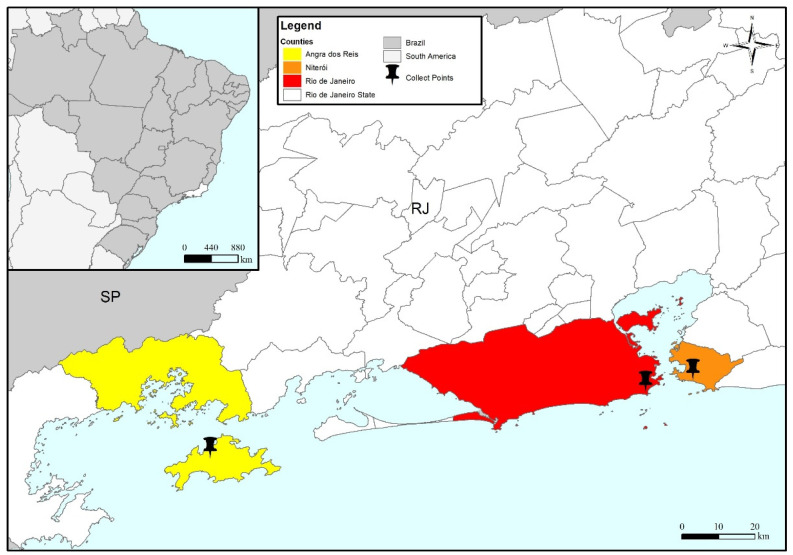
Geographic map of Brazil, highlighting the state of Rio de Janeiro, with the three collected points evaluated in this study: Angra dos Reis (yellow), Niterói (orange), and Rio de Janeiro (red) cities from January to December 2022.

Mussels (*Perna perna* and *Perna viridis*) and oysters (*Crassostrea gigas* and *Crassostrea gasar*) were biweekly purchased at one producer and two local markets in three cities in Rio de Janeiro state, Brazil: Angra dos Reis, Rio de Janeiro, and Niterói, respectively (Figure 1), from January to December 2022. The bivalve samples were prepared as described in the ISO/TS 15216-1:2017 [34]. The mengovirus (MgV vMC_0_), a non-virulent murine mutant strain lacking the poly(C) tract and a member of the *Picornaviridae* family, was used as an internal extraction process control. Previously, MgV vMC_0_ was propagated in sub-confluent VERO cells (ATCC-CCL-81), as described by Cantelli et al. [35]. Ten microliters of MgV vMC_0_ (7.7 × 10^6^ genome copies (GC)/µL) were spiked in each bivalve sample (a pool of 12–15 digestive glands was dissected) and homogenized for 3 min in a vortex with 2 mL of a proteinase K solution (100 µg/mL). This mixture was incubated at 37 °C with shaking (250 rpm) for 70 min, followed by incubation at 60 °C for 20 min, and centrifugation at 3000× *g* for 5 min was performed. The supernatants recovered were immediately processed or stored at −80 °C.

### 2.2. Nucleic Acid Extraction and Viruses Detection

The total nucleic acid extraction from 300 µL of the viral concentrate was carried out using the Chemagic Viral DNA/RNA 300 H96 kit at the Janus^®^G3/Chemagic^TM^360 Workstation (PerkinElmer, Waltham, MA, USA), according to the manufacturer’s instructions. Nucleic acids were eluted into 65 µL of the elution buffer and kept at −80 °C until biomolecular analysis.

Gastroenteric viruses were detected and quantified using the RT-qPCR or qPCR TaqMan^®^ system (ABI PRISM 7500^®^, Applied Biosystems). The amplification conditions and the set of primers and probes (10 pmol of each) used are described in Table 1. Molecular reactions were performed using the SuperScript™ III Platinum™ One-Step qRT-PCR Kit (ThermoFisher Scientific, Invitrogen Division, Carlsbad, CA, USA) for noroviruses GI/GII, RVA, SaV, and MgV vMC_0_ and the TaqMan Universal Master Mix kit (Applied Biosystems, Foster City, CA, USA) for the human mastadenovirus in a 25 µL final volume (5 µL of nucleic acid viral). Positive controls (containing RNA extracted from virus suspensions) and negative controls (DNAse and RNAse-free water) were included in all qPCR assays. The viral nucleic acid obtained from each bivalve sample was tested, pure and 10-fold-diluted in duplicate, to evaluate possible inhibitor interference, totaling four qPCR reactions per sample. The standard curves were generated using 10-fold serial dilutions of a double-stranded DNA fragment containing the amplification region sequence of each virus (gBlock Gene Fragment, Integrated DNA Technologies^®^, Coralville, IA, USA). The slope ranged from −3.311 to −3.512, the square regression coefficient (r^2^) value was 0.998–1.000, and the reaction efficiencies were 91.68–103.5%. For each tested virus, the samples were considered positive when at least the duplicate analyzed (pure or 10-fold-diluted) was detected at the cycle threshold (Ct) ≤ 42, showing a characteristic sigmoid curve. MgV vMC_0_ recovery rate percentages were calculated for each average replicate sample (pure and 10-fold-diluted), and a >1% extraction efficiency was regarded as a successful extraction of nucleic acid from the samples as described in ISO/TS 15216:2017 [34].

### 2.3. Molecular Typing of Gastroenteric Viruses

For RNA viruses (noroviruses GI/GII, RVA, and SaV), the synthesis of complementary DNA (cDNA) was performed with a total volume of 50 µL using a High-Capacity cDNA Reverse Transcription Kit (Applied Biosystems^®^, Foster City, CA, USA). Previously, the reaction mixture comprising 15 µL of RNA and 5 µL of a 10X RT Random Primer was heated at 97 °C for 7 min to facilitate the opening of the RNA secondary structure. Next, the reaction was incubated with 30 µL of the mix containing 21 µL of water RNase and DNase-free, 2 µL of the 25X dNTP mix (100 mM), 5 µL of the 10X RT buffer, and 2 µL of MultiScribe Reverse Transcriptase (50 U/μL). The amplification conditions were performed according to the manufacturer’s instructions.

PCR rounds were performed using 50 µL of a reaction mixture containing 10 µL of cDNA (for RNA virus) or 10 µL of DNA (human mastadenovirus)—the 1st round—and from 5 µL (RVA) to 10 µL (norovirus, SaV) of the 1st amplicon in 2nd round, using Platinum™ Taq DNA Polymerase (Invitrogen^®^) in PCR and semi-nested PCR assays, respectively, according to the manufacturer’s instructions. The amplification conditions and the set of primers (25 pmol of each, except norovirus at 50 pmol) used are described in Table 2. All assays included negative and positive reaction controls. Seven microliters of each PCR reaction were analyzed by electrophoresis in 1.2% agarose gel at 100 V for 45 min. The generated amplicons of norovirus GII (390 base pairs (bp)), noroviruses GI (380-pb), VP7 RVA (881-pb), VP8* RVA (663-pb), SaV (380-pb), and human mastadenovirus (301-bp) were excised from the gel and purified using Wizard^®^ SV Gel and a PCR Clean-Up System kit (Promega, Madison, USA) following the manufacturer’s instructions. The genotyping of human mastadenoviru and RNA viruses was performed by a PCR and semi-nested PCR followed by the Sanger sequencing of the first and second round PCR amplicons, respectively, using a BigDye^®^ Terminator v3.1 Cycle Sequencing Kit and an ABI Prism 3730xl Genetic Analyser^®^ (Applied Biosystems, Foster City, CA, USA) via the FIOCRUZ Institutional Sequencing Platform (PDTIS). Consensus sequences were obtained using Geneious Prime 2021.1.1 software (Biomatters Ltd., Auckland, New Zealand), and nucleotide similarity was compared with the available information assessed using the Basic Local Alignment Search Tool (https://blast.ncbi.nlm.nih.gov/Blast.cgi, accessed on 5 November 2023). Norovirus genotypes were assigned using the two norovirus typing tools (https://www.rivm.nl/mpf/typingtool/norovirus, accessed on 7 November 2023 and https://norovirus.ng.philab.cdc.gov, accessed on 10 November 2023), and RVA genotypes were assigned using rotavirus A genotyping tool (https://www.rivm.nl/mpf/typingtool/rotavirusa/, accessed on 2 December 2023). The sequences obtained in this study were deposited in the GenBank database under the accession numbers OR984191-OR984203 (norovirus GII), PP025339-PP025347 (RVA), PP035205-PP035209 (human mastadenovirus), PP035210-PP035211 (SaV). Phylogenetic analyses were performed using the maximum-likelihood method, with the Kimura 2-parameter model applied for the analysis of the regions evaluated (norovirus GII—region C/5′ ORF-2; RVA—genes VP7 and VP8*; SaV—gene VP1; and human mastadenovirus—*Hexon* gene). Phylogenetic trees were constructed with 2000 bootstrap replicates using MEGA software version 11.

### 2.4. Statistical Analysis

Descriptive statistical analyses were performed using GraphPad Prism version 9.0.0^®^ (GraphPad Software^®^, San Diego, CA, USA). Viral concentrations (DNA or RNA genome copies/g) recovered in bivalve samples were analyzed for significant differences using the independent samples Mann–Whitney U Test. Box-and-whisker plots were calculated to show the differences between medians. The results were considered statistically significant when the *p*-level ≤ 0.05.

## 3. Results

### 3.1. Gastroenteric Viruses’ Detection in Commercial Bivalves

Over the 12-month study period, 134 commercial bivalve samples (72 oysters and 62 mussels) were obtained biweekly from three Rio de Janeiro state cities. Of the 134 bivalves, 50 were collected from a local producer in Angra dos Reis city, and 47 and 37 were collected from local fresh markets in Niterói and Rio de Janeiro, respectively. In some cases, oysters or mussels were not available at the time of purchase, so the number of samples collected varied (average 11 samples per month). The extraction efficiency of all processed pooled samples was calculated based on MgV vMC_0_ recovery. MgV vMC_0_ was detected in 100% of the samples analyzed (*n* = 134), and the recovery rate was 27% ± 20.9 (mean ± standard deviation (sd). All bivalve samples were screened for norovirus (GI and GII), SaV, RVA, and human mastadenovirus.

**Figure 2:**
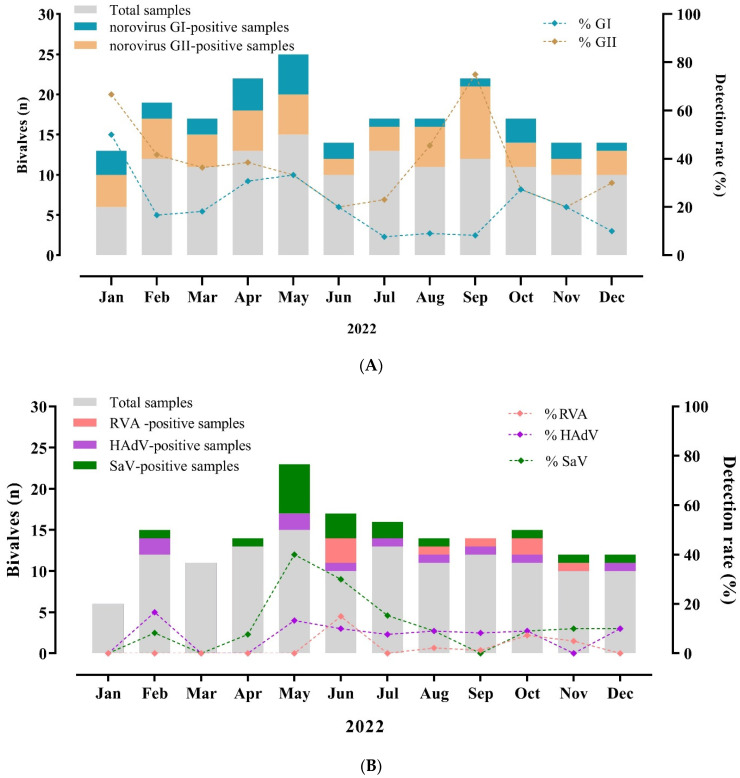
Distribution and percentage of norovirus GI and GII (**A**), rotavirus A (RVA), human mastadenovirus (HAdV), and sapovirus (SaV) (**B**) detected in commercial bivalve mollusks collected over a 12-month period—January to December 2022—from three sampling sites: Angra dos Reis, Niterói, and Rio de Janeiro cities, Brazil.

Noroviruses were the most frequently detected in 40.3% (54/134) samples year-round. Among these, noroviruses GI and GII were detected in 20.1% (27/134) and 37.3% (50/134), respectively (Table 2). The detection rates of noroviruses varied monthly from 8.3 to 50% for GI and from 20 to 75% for GII (Figure 2A). Norovirus GII and GI-positive samples showed viral loads ranging from 1.5 × 10^2^ to 1.3 × 10^7^ CG/g (mean of 3.4 × 10^5^ CG/g) and from 4.9 × 10^1^ to 3.9 × 10^3^ CG/g (mean of 6.7 × 10^2^ CG/g), respectively. SaV was the second most frequently detected virus, with a positivity rate of 12.7% (17/134), and the higher detection rates were between May and July 2022 (autumn and winter) (Figure 2B). For SaV-positive samples, viral loads varied from 1.1 × 10^2^ to 1.1 × 10^5^ CG/g (mean of 1.4 × 10^4^ CG/g).

**Figure 3:**
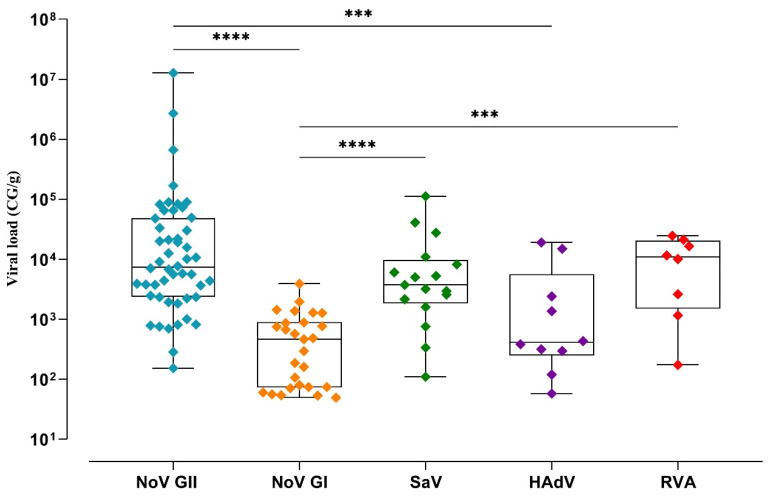
Norovirus (NoV) GII, NoV GI, sapovirus (SaV), human mastadenovirus (HAdV), and rotavirus A (RVA) viral loads in bivalves collected over a 12-month period—January to December 2022—from three sampling sites: Angra dos Reis, Niterói, and Rio de Janeiro cities, Brazil. Box and whisker plots showing all the values distributed within the median (horizontal line in the box) and range concentrations (genome copies per gram (GC/g) of digestive tissue). *** *p* < 0.001; **** *p* < 0.0001.

Human mastadenovirus and RVA were detected less frequently, with detection rates of 7.5% and 5.9%, and viral loads varied from 5.8 × 10^1^ to 1.9 × 10^4^ CG/g (mean of 4 × 10^3^ CG/g) and from 1.7 × 10^2^ to 2.5 × 10^4^ CG/g (mean of 1.1 × 10^4^ CG/ g), respectively. The human mastadenovirus did not display a seasonal pattern, and RVA was detected more frequently in the winter and spring seasons (Figure 2B). Analyzing the viral loads between the gastroenteric viruses observed a statistically significant difference between norovirus GII compared to GI (*p* ≤ 0.0001) and human mastadenovirus (*p* = 0.0009), and among norovirus GI, compared to SaV (*p* ≤ 0.0001) and RVA (*p* = 0.0003) (Figure 3).

Rio de Janeiro reported higher norovirus detection (62.2%) (Table 3) and the majority of single and co-detections compared to other sites (22/60) (Table 4). In total, 44.8% (60/134) of the bivalve samples tested positive for one or more viruses, with 46.7% (28/60) of positive samples testing positive for a single viral agent, 26.7% (16/60) for two viral agents, 8.3% (5/60) for three viral agents, and 13.3% (8/60) for four viral agents. Three mussel samples were also contaminated with the five gastroenteric viruses investigated (5%, 3/60) (Table 5).

### 3.2. Gastroenteric Viruses Genotyping

**Figure 4:**
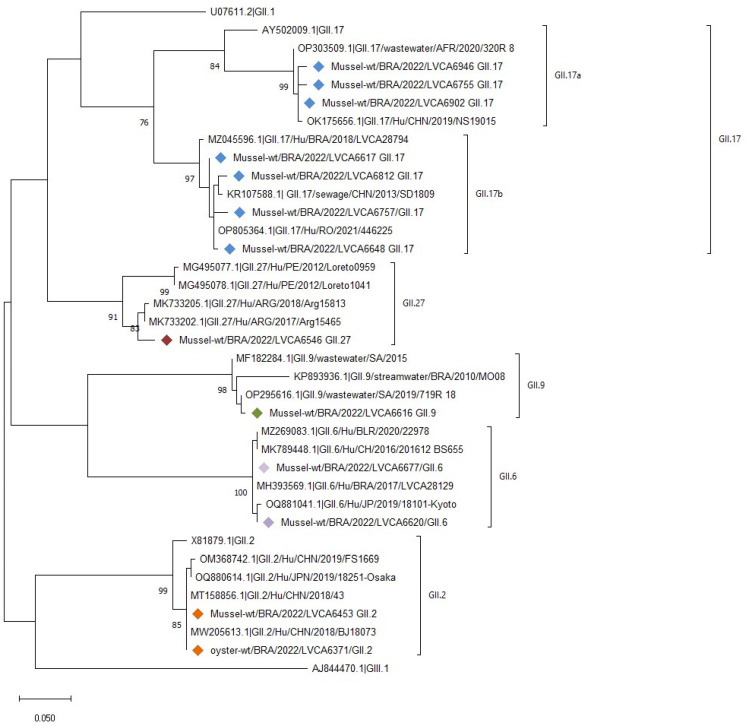
Phylogenetic tree based on the partial capsid (ORF2, VP1 gene) region of norovirus GII. References strains were downloaded from GenBank and labeled with their respective accession numbers. Sequences obtained from bivalve samples (marked with a diamond) in this study from January to December 2022 in Angra dos Reis, Niterói, and Rio de Janeiro cities are shown per country followed by the internal register code number plus the city’s location, year of collection, and genotype (i.e., BRA/LVCA6546_city of Rio de Janeiro/2022/GII.27). Maximum likelihood phylogenetic trees were constructed with MEGA 11 software and bootstrap tests (2000 replicates) based on the Kimura 2-parameter model. The bootstrap percentage values of >70 are shown.

Regarding norovirus GII characterization, 48.1% (13/27) of the positive samples selected (Ct < 37) were successfully sequenced. The most predominant genotype found during monitoring was GII.17 (53.8%, n = 7), followed by GII.2 (15.4%; n = 2), GII.6 (15.4%; n = 2), GII.9 (7.7%, n = 1), and GII.27 (7.7%; n = 1) (Figure 4). GII.17 norovirus strains were segregated into two different clusters as follows: GII.17a sublineage (n = 3) circulated in three cities in the Rio de Janeiro state, showing a high nucleotide (nt) identity (99%) with a strain detected in wastewater in Africa, 2020 (OP303509); the GII.17b sublineage (n = 4) shared 97% of nt identity with strains detected in sewage in China, 2013 (KR107588) and clinical sample in Brazil, 2018 (MZ045596). The GII.2 genotype (n = 2) detected in Angra dos Reis shared 100% nt identity with clinical strains from China (OM368742 and MT158856, 2018–2019) and Japan (OQ880614, 2019). The GII.27 genotype detected in Rio de Janeiro’s municipality was similar to those that were first described in South America in Peru (MG495077 and MG495078) (Figure 4), sharing high nt similarities with sequences described in Argentina during 2017–2018 (MK733202 and MK733205). Concerning norovirus GI, 25% (2/8) positive samples (CT < 38) were amplified. However, the electropherograms did not yield a satisfactory resolution. The chromatograms show the mixed bases in some positions (equal intensities), indicating that *quasispecies* might be present (data not shown).

**Figure 5:**
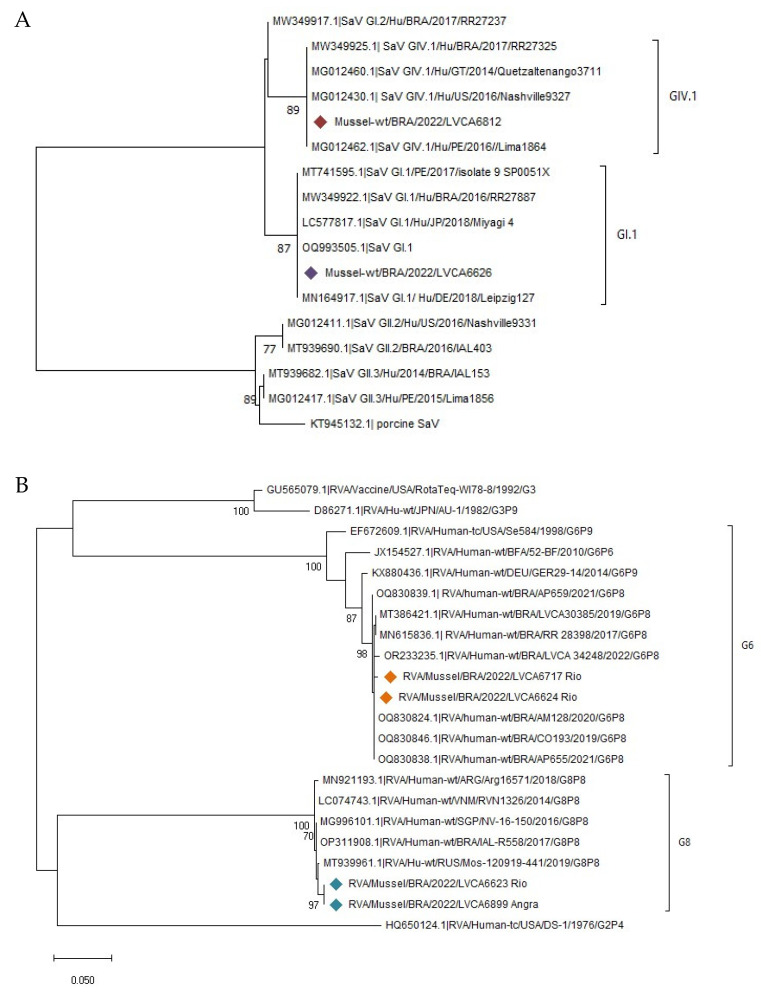

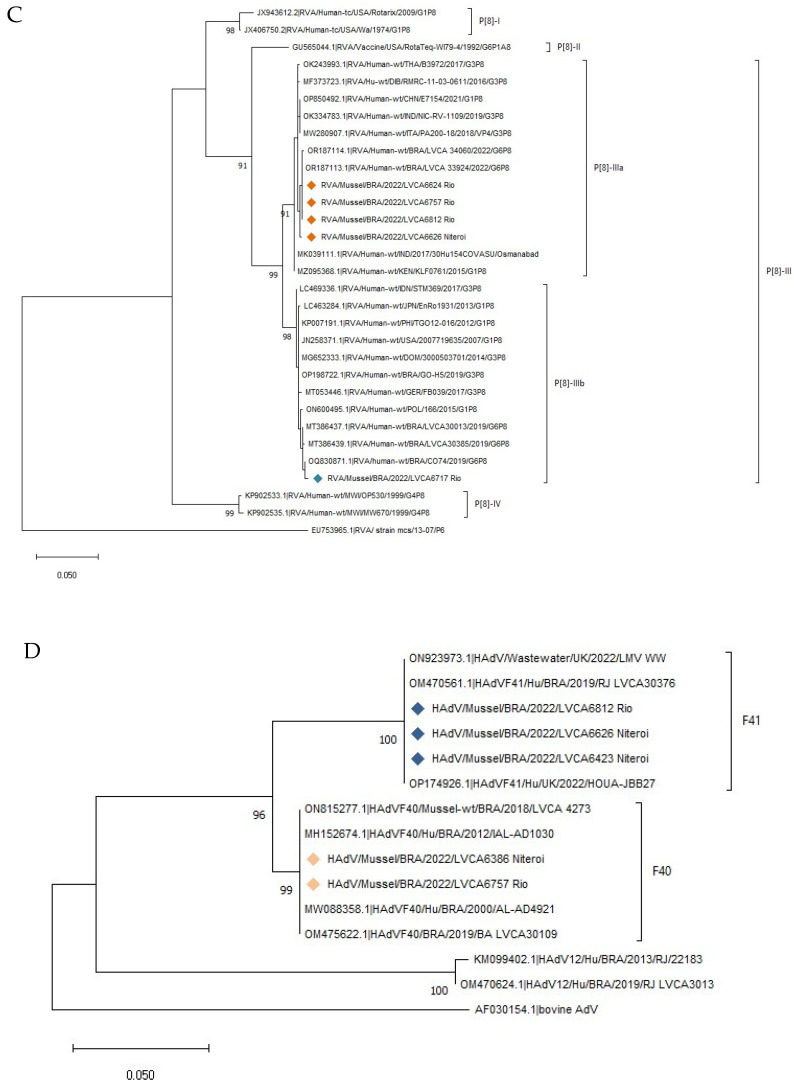
Phylogenetic trees based on (**A**) the partial VP1 gene of the sapovirus (SaV); (**B**) the partial VP7 gene of rotavirus A (RVA); (**C**) the partial VP4 region of (RVA), and (**D**) the *hexon* gene of the human mastadenovirus (HAdV). References strains were downloaded from GenBank and labeled with their respective accession numbers. Sequences obtained from bivalve samples (marked with a diamond) in this study from January to December 2022 in Angra dos Reis, Niterói, and Rio de Janeiro cities are shown per country followed by the internal register code number plus the city’s location, and year of collection (i.e., BRA/LVCA6812_city of Rio de Janeiro/2022). Maximum likelihood phylogenetic trees were constructed with MEGA 11 software and bootstrap tests (2000 replicates) based on the Kimura 2-parameter model. The bootstrap percentage values of >70 are shown.

To analyze SaV, eight positive samples with relatively lower Ct values (Ct < 38) were chosen; only 25% (2/8) of them could be amplified to examine the genotypes that spread in the cities of Niterói and Rio de Janeiro in 2022. The GI.1 and GIV.1 observed showed an 87% to 89% nucleotide identity, respectively, compared to the reference strains originating in Brazil, the USA, Peru, Guatemala, Nicaragua, and Japan (Figure 5A).

RVA was detected in bivalves with a detection rate of 5.9%, and the two partial genes of viral capsid VP7 (G) and VP8* (P) were investigated. The phylogenetic analysis of the partial VP7 nucleotide sequences of four RVA isolates detected in mussels revealed G6 and G8 genotypes circulating in the city of Rio de Janeiro; the genotype G6 (two samples) showed high similarity with clinical samples from Brazil (2017–2022), while the G8 genotype (two samples) was closely related to samples from Brazil, Singapore, Russia, and Argentina (2014–2019) (Figure 5B). Regarding the VP8* gene, the circulating genotype was P[8]-III and segregated into two different clusters with nucleotide similarities ranging from 91% to 98%, and sequences from Brazil, Italy, Japan, India, Germany, and Thailand, between 2015 and 2022 (Figure 5C).

Regarding the human mastadenovirus, the partially conserved region of the *hexon* gene could be characterized in 50% (5/10) of positive samples. The enteric genotypes F40 and F41 were detected, showing high similarities with clinical samples in Brazil (OM470561, OM475622, MH152674) and the United Kingdom (UK) in 2022 (OP174926), and environmental samples detected in Brazil in 2019 (mussel, ON815277) and in the UK in 2022 (sewage, ON923973) (Figure 5D).

## 4. Discussion

Considering the lack of information and the risk of food consumption in Brazil, our one-year study provides evidence and information on the occurrence of gastroenteric viruses in bivalve mollusks in the tourist cities of Rio de Janeiro. It contributes to understanding the potential menace of the occurrence and diversity of gastroenteric viruses detected from commercialized bivalves collected in three tourist coastal cities of Rio de Janeiro, Brazil. Noroviruses showed the highest detection rate year-round (40.3%); similarly, frequency patterns for noroviruses were reported by other recent studies in Brazil (41.6%, [46]), Italy (39–43%, [47,48,49,50]), and Montenegro (43%, [51]).

Globally, noroviruses are recognized to be the most common cause of foodborne illnesses across all ages in either sporadic AGE cases or outbreaks [52,53,54] and represent serious public health risks [55,56,57]. Besides this, noroviruses appear as one of the most frequent human viruses in bivalves worldwide and have been detected with varying prevalences (0–91.7%) [2,12,58,59].

Concerning norovirus GII concentration, our results were similar to those obtained in oysters collected from seafood markets in China (10^3^–10^6^ GC/g) [60]. According to Teunis et al. [61], fewer than 10 to 100 virus particles can be infectious doses for norovirus GII and human mastadenovirus, respectively; thus, a low viral load of norovirus GII in bivalves may pose a potential risk of AGE [62,63].

The recognition of bivalves as a source of infectious disease hazards led to the establishment of sanitary controls in most countries for their production by classifying the harvesting areas or their commercialization [64]. These areas are classified according to *Escherichia coli* levels, but their use as the sole bioindicator of fecal contamination may not represent a direct correlation with gastroenteric viruses [12,65,66,67,68]. The epidemiological surveillance on bivalves has been reported only in specific countries, periods, and bivalve mollusk species analyzed. There is a need for additional data in several regions of the world, mainly Africa and South and Central America, where few studies have been conducted on viral contaminants in bivalves on a limited set of human viruses, which do not reflect the global situation [2,69]. Over the last few years, studies have reported bivalve contamination for enteric viruses in different sites on the Brazilian Southeastern coast, such as the estuary–lagoon complex of Cananéia, São Paulo [70,71], the mangrove estuary in the Vitória Bay region, Espírito Santo [72], and Arraial do Cabo, Rio de Janeiro [46,73], in relation to environmental studies.

Bivalves are known as “hotspots” for the accumulation of multiple norovirus strains [74], presenting opportunities for human co-infection and subsequent viral recombination and are, thus, reservoirs for the introduction of novel recombinant strains into the human population [21]. Recently, norovirus GII.17[P17] and GII.2[P16] genotypes emerged as a significant cause of AGE outbreaks, indicating an increase in genotypic diversity [75,76,77,78]. Oysters and other bivalves have been suggested as common vehicles for the transmission of emerging GII.17 viruses [79,80]. Rasmussen et al. [81] could establish a direct molecular link between imported oysters acting as vehicles for a series of AGE outbreaks for GII.17 in Denmark and France in 2016. In our study, GII.17 was the most predominant genotype found (53.8%) during the bivalve monitoring in Rio de Janeiro. Fumian et al. [82] reported GII.17[P17] as the predominant genotype detected (45.5%) in clinical samples associated with an AGE outbreak after storm events in Santa Catarina, Southern Brazil. The finding of the GII.17[P17] genotype detected in diarrheal and non-diarrheal cases in a pediatric cohort [83] suggests that this genotype has circulated in the Brazilian population since 2015, in addition to that associated with foodborne transmission, according to reports by da Silva Ribeiro de Andrade et al. [84].

The first evidence of the genotype GII.27, which is partially characterized by the VP1 gene, appeared in Brazil. GII.27 was considered a non-typeable GII, first described in Peru in 2010 (MK733206-MK733207) and 2012 (MG495077-MG495078) associated with two polymerase genotypes (GII.[P26] and GII.[P27]), and in Argentina between 2017 and 2018 (MK733202-MK733205), it was associated with the GII.[PNA9] genotype, totalizing eight complete sequences to date (https://calicivirustypingtool.cdc.gov/gebali.cgi?GII.27, accessed on 10 November 2023). According to Tohma et al. [85], the limited detection of this GII.27 genotype in South America could result from some factors such as low transmissibility, host-related susceptibility, or underreporting because of limited noroviruses surveillance or nucleotide mismatches in the PCR oligos for detection.

Besides that, the present study highlights SaV GI.1 and GIV.1 detection in bivalves, which is, to date, the first monitoring of this virus reported in South America. Globally, few studies have investigated the presence of SaV in bivalves. Between 2002 and 2006, SaV was detected in 3 out of 11 outbreaks that occurred in Japan due to consuming contaminated oysters [86]. In an 18-month survey from class B mollusk-harvesting areas in Northwest Spain, SaV was detected in wild and cultured mussels with a prevalence of 14.3% and 12.9%, respectively, and the genotypes circulating were GI.1, GI.2, GIV.1, and GV.1 [7]. In Southwest Italy, SaV was detected in 18.8% of bivalves from three coastal areas of the Campania region from 2015 to 2017 [48], and 12% in *Crassostrea gigas* evaluated in this study as a model for correlating viral and chemical contamination in the marine environment, performed by Fiorito et al. [50].

Concerning the human mastadenovirus concentration, our results were similar to those obtained in oysters and mussels collected in Vitória Bay in Southeastern Brazil (4.5 × 10^2^–1.2 × 10^3^ GC/g) [72]. However, in Southern Brazil, Rigotto et al. [87] showed that the human mastadenovirus requires loads between 10^4^ and 10^5^ GC/g in oysters collected on different farms. Fusco et al. [48] reported the frequency of 5.6% of human mastadenovirus in bivalve samples collected in coastal areas of the Campania region in Southwestern Italy from 2015 to 2017. In Mumbai, India, the incidence of human mastadenovirus in seafood from different landing centers and markets was 21%, with the highest incidence in clam samples (14.9%) [88]. Besides that, several environmental studies in Brazil have demonstrated high frequencies of the human mastadenovirus in coastal waters, lagoons, and bivalves [73,89,90,91,92]. The predominance of human mastadenovirus F40 and F41 corroborated with other studies, revealing the predominance of these types circulating in clinical samples in Brazil [93,94,95]. Recently, the F41 serotype was detected in wastewater samples in Ireland [96], Australia [97], the UK [98], and Japan [99].

After the COVID-19 pandemic, with the end of restrictive measures, it was observed that the number of positive cases of RVA of the G6P[8] genotype increased considerably in Brazil, reaching 63% of the clinical cases analyzed, especially in the years 2022 and 2023. The data found in this study agree with clinical studies demonstrating the circulation of this genotype [100,101]. G8 genotype strains, also detected in this study, are considered rare or uncommon [102]. Recently, Medeiros et al. [103] suggested that Brazilian bovine-like G8P[8] strains with DS-1-like backbone strains are continuously evolving and probably reassorting with local RVA strains rather than directly related to Asian imports. However, the increase in G8 detection outside Africa may indicate that G8 strains are emerging worldwide and that this genotype should be monitored [104,105].

This study presents some limitations as follows: (i) First, the recovery rates of an external amplification control (target synthetic viral RNA) were not evaluated as suggested by ISO/TS 15216. This parameter is recommended to investigate possible RT-PCR interference by validating recovery rates >25% in pure extracted RNA. (ii) Second, it is possible that the viruses in some positive samples were not detected due to their very low viral loads. According to MgV vMC_0_ recovery rates, it is possible that the extraction method used resulted in a loss of yield (which could result in false negatives). Indeed, the silica-magnetic bead extraction method could likely improve viral recovery efficiency for this analysis. (iii) Finally, considering the sequencing method chosen in this study (Sanger), with advances in next-generation sequencing (NGS) technology, the use of NGS technology could determine the diversity for a more comprehensive understanding of viral epidemiology in commercialized bivalve mollusks. Indeed, for the following surveillance studies, this approach allows possible correlations of genotypic diversity between bivalve/environmental and clinical samples, acting as food safety tools.

## 5. Conclusions

This study highlights the use of bivalve mollusk surveillance to investigate the emergence of viruses in specific geographical areas and provides unprecedented epidemiological surveillance data on commercial bivalves in Brazil. Certainly, viral surveillance can be a tool for quality assessment to improve the production chain of these foods as a food safety measure. Molecular diagnosis, including quantification and genotyping, should be used to understand the burden of virus contamination and the spread of emerging pathogens through viral epidemiological and sanitary surveillance systems concerning the supply of these foods to the population. In addition to food safety measures to raise awareness of infection through consumption, regular surveillance can help reduce global outbreaks through the early detection of emerging viruses.

## Figures and Tables

**Table 1:** Primers and probes used for real-time PCR assays for norovirus GI and GII, rotavirus A, sapovirus, mengovirus, and human mastadenovirus in bivalves collected over 12 months (January to December 2022) from three sampling sites: Angra dos Reis, Niteroi, and Rio de Janeiro cities, Brazil.

Virus Target	Primers and Probes	Sequences (5′–3′)	Partial Region	Reference	Amplification Conditions
Norovirus GI	COG1-F	CGY TGG ATG CGN TTY CA	ORF1/2 junction	[36]	55 °C/30 min 95 °C/10 min X 45 cycles 95 °C/15 s 60 °C/1 min
	COG1-R	CTT AGA CGC CAT CAT CAT TYA C			
	RING1c	^a^HEX-AGA TYG CGR TCY CCT GTC CA			
Norovirus GII	COG2-F	CAR GAR BCN ATG TTY AG			
	COG2-R	TCG ACG CCA TCT TCA TTC ACA			
	RING2	^b^FAM-TGG GAG GGC GAT CGC AA			
Rotavirus A	NSP3-F	ACC ATC TWC ACR TRA CCC TCT ATG AG	NSP3 gene	[37]	
	NSP3-R	GGT CAC ATA ACG CCC CTA TAG C			
	NSP3-P	^a^HEX-AGT TAA AAG CTA ACA CTG TCA AA-MGB-NFQ^c^			
Sapovirus	HuSaV-F1	GGC HCT YGC CAC CTA YAA YG	VP1 gene	[38]	
	HuSaV-F2	GAC CAR GCH CTC GCY ACC TAY GA			
	HuSaV-F3	GCW RYK GCW TGY TAY AAC AGC			
	HuSaV-R	CCY TCC ATY TCA AAC ACT A			
	HuSaV-TP-a	^b^FAM-CCN CCW ATR WAC CA-MGB-NFQ^c^			
	HuSaV-TP-b	^b^FAM-CCN CCW ACR WAC CA-MGB-NFQ ^c^			
Mengovirus	Mengo110-F	GCG GGT CCT GCC GAA AGT	5′-NCR	[39]	
	Mengo209-R	GAA GTA ACA TAT AGA CAG ACG CAC AC			
	Mengo147	^b^FAM-ATC ACA TTA CTG GCC GAA GC			
Human mastadenovirus	Ad-F	C(AT)T ACA TGC ACA TC(GT) C(CG)G G	*Hexon* gene	[40]	50° C/2 min 95 °C/10 min X 45 cycles 95 °C/15 s 60 °C/1 min
	Ad-R	C(AG)C GGG C(GA)A A(CT)T GCA CCA G			
	Ad-P	^b^FAM-CCG GGC TCA GGT ACT CCG AGG CGT CCT			

IUPAC codes indicate degenerate positions; ^a^ HEX: hexachlorofluorescein reporter dye; ^b^ FAM: 6-carboxyfluorescein reporter dye; and ^c^ MGB: minor groove binder.

**Table 2:** Set of primers used to type PCR assays for norovirus GI and GII, rotavirus A, sapovirus, and human mastadenovirus in bivalves collected over 12 months (January to December 2022) from three sampling sites: Angra dos Reis, Niterói, and Rio de Janeiro cities, Brazil.

Virus Target	Primers	PCR Round	Sequences (5′–3′)	Partial Region	References	Amplification Conditions For 1st and 2nd Rounds
Norovirus GI	Mon432-F	1st	TGG ACI CGY GGI CCY AAY CA	region C (ORF-2)	[36,41,42]	94 °C/3 min X 45 cycles (94 °C/30 s, 55 °C/30 s, 72° C/1 min) 72° C/7 min
	G1-SKR	1st, 2nd	CTG CCC GAA TTY GTA AAT GA			
	COG1-F	2nd	CGY TGG ATG CGN TTY CAT GA			
Norovirus GII	Mon431-F	1st	TGG ACI AGR GGI CCY AAY CA			
	G2-SKR	1st, 2nd	CCR CCN GCA TRH CCR TTR TAC AT			
	COG2-F	2nd	CAR GAR BCN ATG TTY AGR TGG ATG AG			
Rotavirus A	9con1-F	1st	TAG CTC CTT TTA ATG TAT GG	VP7 gene	[43]	94 °C/10 min X 45 cycles (94 °C/30 s, 50 °C/30 s 72° C/1 min) 72° C/7 min
	9con2R	1st	GTA TAA AAT ACT TGC CAC CA			
	VP7-F	2nd	ATG TAT GGT ATT GAA TAT ACC AC			
	VP7-R	2nd	AAC TTG CCA CCA TTT TTT CC			
	F-con3	1st	TGG CTT CGC TCA TTT ATA GAC A	VP4 gene (VP8*)		
	R-con2	1st	ATT TCG GAC CAT TTA TAA CC			
	VP4-F	2nd	TAT GCT CCA GTN AAT TGG			
	VP4-R	2nd	ATT GCA TTT CTT TCC ATA ATG			
Sapovirus	M13F-HuSaV-F1	1st	tgtaaaacgacggccagt GGC HCT YGC CAC CTA YAA YG	VP1 gene	[44]	94 °C/3 min X 50 cycles (94 °C/30 s, 55 °C/30 s, 72° C/1 min) 72° C/7 min
	M13F-HuSaV-F2	1st	tgtaaaacgacggccagt GAC CAR GCH CTC GCY ACC TAY GA			
	M13F-HuSaV-F3	1st	tgtaaaacgacggccagt GCW RYK GCW TGY TAY AAC AGC			
	M13R-HuSaV-5498-R	1st, 2nd	caggaaacagctatgacc CCC CAN CCN GCV HAC AT			
	M13F-HuSaV-5159-F	2nd	tgtaaaacgacggccagt TAG TGT TTG ARA TGG ARG G			
Human mastadenovirus	Hex1deg-F	1st	GCC SCA RTG GKC WTA CAT GCA CAT C	*Hexon* gene	[45]	94 °C/3 min X 45 cycles (94 °C/ 30 s, 55 °C/30 s, 72° C/1 min) 72° C/7 min
	Hex2deg-R	1st	CAG CAC SCC ICG RAT GTC AAA			

IUPAC codes are used to indicate degenerate positions.

**Table 3:** Prevalence of bivalve contamination for each viral target * in bivalves collected over 12 months (January to December 2022) from three sampling sites: Angra dos Reis, Niteroi, and Rio de Janeiro cities, Brazil.

Bivalves	Samples (*n*)	Norovirus Detection (%)				Other Viruses Detected (%)			
		GI	GII	GI + GII	Total	SaV	HAdV	RVA	Total
Oysters	72	1 (1.4)	6 (8.3)	2 (2.8)	9 (12.5)	2 (2.8)	1 (1.4)	2 (2.8)	5 (6.9)
Mussels	62	3 (4.8)	21 (33.9)	21 (33.9)	45 (72.6)	15 (24.2)	9 (14.5)	6 (9.7)	30 (48.4)
Total	134	4 (2.9)	27 (20.1)	23 (17.2)	54 (40.3)	17 (12.7)	10 (7.5)	8 (5.9)	35 (26.1)

* norovirus GI, norovirus GII, sapovirus (SaV), human mastadenovirus (HAdV), and rotavirus A (RVA).

**Table 4:** Norovirus detection frequency from bivalve samples during the 12-month (January to December 2022) monitoring period in Rio de Janeiro, Brazil.

City	Samples	Detection (%)	Genogroup Identified	*p-*Level
Angra dos Reis	50	17 (34)	GI, GII, GI + GII	Angra vs. Niterói (*p*: 0.8349)
Niterói	47	17 (36.2)	GII, GI + GII	Rio vs. Niterói (*p*: 0.0274)
Rio de Janeiro	37	23 (62.2)	GI, GII, GI + GII	Rio *vs* Angra (*p*: 0.0162)

**Table 5:** Detections (single and multiple) of gastroenteric viruses * by commercialized bivalve species and collection cities in Rio de Janeiro, Brazil, during the 12-month (January to December 2022) monitoring period.

Bivalve	Site	Samples (*n*)	Number of Positive Samples														
			Single Detection				Double Detection					Triple Detection		Quadruple Detection		Quintuple Detection	
			NoV GII	NoV GI	HAdV	RVA	NoV GII + GI	NoV GII + SaV	NoV GI + SaV	SaV + RVA	NoV GI + RVA	NoV GI + GII + SaV	NoV GI + GII + HAdV	NoV GI + GII + SaV + HAdV	NoV GI + GII + HAdV + RVA	NoV GI + GII + SaV + HAdV + RVA	Total
	Angra	25	**5**	**1**	0	**1**	**2**	0	**1**	0	**1**	0	0	0	0	0	11
Mussels	Rio	22	**7**	0	0	0	**4**	**2**	0	0	0	**2**	0	**4**	**1**	**2**	**22**
	Niteroi	15	**6**	0	0	0	**2**	**1**	0	0	0	**2**	**1**	**3**	0	**1**	16
	Angra	25	**2**	0	0	0	0	0	0	0	0	0	0	0	0	0	2
Oysters	Rio	25	**2**	**1**	**1**	0	**1**	0	0	**1**	0	0	0	0	0	0	**6**
	Niterói	22	**1**	0	0	**1**	0	**1**	0	0	0	0	0	0	0	0	3
	Total	134	**23**	**2**	**1**	**2**	**9**	**4**	**1**	**1**	**1**	**4**	**1**	**7**	**1**	**3**	**60**

* norovirus GI (NoV GI), norovirus GII (NoV GII), sapovirus (SaV), human mastadenovirus (HAdV), and rotavirus A (RVA).

## Data Availability

Data will be made available on request.
